# Application of Dominance-Based Rough Set Approach for Optimization of Pellets Tableting Process

**DOI:** 10.3390/pharmaceutics12111024

**Published:** 2020-10-26

**Authors:** Maciej Karolak, Łukasz Pałkowski, Bartłomiej Kubiak, Jerzy Błaszczyński, Rafał Łunio, Wiesław Sawicki, Roman Słowiński, Jerzy Krysiński

**Affiliations:** 1 Department of Pharmaceutical Technology, Collegium Medicum, Nicolaus Copernicus University, 85-089 Bydgoszcz, Poland; lukaszpalkowski@cm.umk.pl (Ł.P.); jerzy.krysinski@cm.umk.pl (J.K.); 2 Adamed Pharma S.A., Pieńków, 05-152 Czosnów, Poland; bartlomiej.kubiak@adamed.com.pl; 3 Institute of Computing Science, Poznań University of Technology, 60-965 Poznań, Poland; jerzy.blaszczynski@cs.put.poznan.pl (J.B.); roman.slowinski@cs.put.poznan.pl (R.S.); 4 Polpharma SA, 83-200 Starogard Gdański, Poland; rafal.lunio@polpharma.com; 5 Department of Physical Chemistry, Medical University of Gdańsk, 80-416 Gdańsk, Poland; wieslaw.sawicki@gumed.edu.pl; 6 Systems Research Institute, Polish Academy of Sciences, 01-447 Warsaw, Poland

**Keywords:** tablets, multiple-unit pellet system, pharmaceutical technology, DRSA, data mining, knowledge discovery, machine learning

## Abstract

Multiple-unit pellet systems (MUPS) offer many advantages over conventional solid dosage forms both for the manufacturers and patients. Coated pellets can be efficiently compressed into MUPS in classic tableting process and enable controlled release of active pharmaceutical ingredient (APIs). For patients MUPS are divisible without affecting drug release and convenient to swallow. However, maintaining API release profile during the compression process can be a challenge. The aim of this work was to explore and discover relationships between data describing: composition, properties, process parameters (condition attributes) and quality (decision attribute, expressed as similarity factor f_2_) of MUPS containing pellets with verapamil hydrochloride as API, by applying a dominance-based rough ret approach (DRSA) mathematical data mining technique. DRSA generated decision rules representing cause–effect relationships between condition attributes and decision attribute. Similar API release profiles from pellets before and after tableting can be ensured by proper polymer coating (Eudragit^®^ NE, absence of ethyl cellulose), compression force higher than 6 kN, microcrystalline cellulose (Avicel^®^ 102) as excipient and tablet hardness ≥42.4 N. DRSA can be useful for analysis of complex technological data. Decision rules with high values of confirmation measures can help technologist in optimal formulation development.

## 1. Introduction

Pellets, microcapsules, as well as other solid particles can form multiple-unit dosage forms. By a proper modification, such forms allow a modified or constant release rate, whereby the concentration of the drug is maintained within therapeutic limits for a longer period of time. An appropriate release profile allows controlled absorption of active pharmaceutical ingredients (APIs) in the specific part of the gastrointestinal tract, providing a desirable therapeutic effect and reduces side effects. A special type of multiparticulate drug delivery systems is a multiple unit pellet system (MUPS) in which microparticles or pellets are compressed to obtain a tablet. Tableted pellets demonstrate faster passage through the esophagus in comparison to capsules, and are more physically stable in comparison to suspensions. MUPS also allow the masking of the taste of active substances, the obtainment of enteric tablets for APIs sensitive to low pH, and the ability to modify or control the release of orally disintegrating dosage forms for geriatric or pediatric patients [1,2].

Tableting of pellets is a major technological challenge. Pharmaceutical technology aims at obtaining MUPS from which pharmacologically active substances are released to obtain an optimal therapeutic effect. In the course of compression, the structure of the pellet shell may be disturbed, which would change the release of the active substance [3]. The deformation of the pellets during tableting depends on many factors, e.g., thickness and type of the coating, properties and structure of pellets core, and composition of a tablet mass. The quality of MUPS is also influenced by tableting process parameters, such as compression force or type of tablet press [4].

For several years, International Conference on Harmonization (ICH) and Food and Drug Administration (FDA) have been promoting the Quality by Design (QbD) approach in pharmaceutical development [5]. One of the objectives of QbD is to ensure the quality of the product by identifying and linking critical material attributes (CMA) and critical process parameters (CPP). CMAs are termed as a physical, chemical or biological property or characteristic of an input material that should be within an appropriate limit, range, or distribution to ensure the desired quality of that drug formulation. At each stage of the product development using QbD, the Design of Experiment (DoE) can be applied. DoE statistical tools reveal relationships between input factors and output responses, and allow to systematically manipulate factors according to a prespecified design. DoE can help to identify optimal conditions, CMAs or CPPs [6].

In order to obtain a proper release of the active substance from MUPS, a number of time-consuming tests and trials have to be carried out. The tests concern the physical and chemical properties of the active substance, the quantity and quality of excipients, and the use of proper technological parameters. The large number of results and factors affecting the quality of the product often makes it difficult to assess and draw conclusions [7], therefore, application of data analyzing tools in order to discover dependencies between the description of the technological process and its result can lead to successful and knowledge-based product development.

In this paper, we present an application of a knowledge discovery technique, called dominance-based rough set approach (DRSA). The presented method is based on rough set theory (RST). It was chosen as the most suitable method to discover synthetic rules that in an intelligible way exhibit monotonic relationships between tableting process parameters and composition of tablets with pellets, and their API release profiles. It handles qualitative and quantitative attributes, without the need of discretization of quantitative-numerical attributes or transformation of qualitative attributes into quantitative-numerical ones. DRSA is also able to deal with possible inconsistencies in data prior to the induction of rules. Moreover, it handles global or local monotonic relationships between values of condition attributes and the quality classes [8]. Using this method, we obtain decision rules with values of condition attributes concerning tablets with pellets.

The aim of this study was to search and discover dependences occurring between technological data describing the composition, properties and process of formulation of tablets with floating pellets and their quality expressed as similarity in APIs release profile before and after tableting. Data analysis was performed using DRSA, which is a novel method in pharmaceutical technology.

## 2. Materials and Methods

### 2.1. Data Set

Data for the analysis describing the parameters of pellets tableting process, tablet mass composition and tablet properties come from the research of Sawicki’s team [9,10,11,12,13,14]. In these studies, the influence of various excipients and technological parameters on the process of obtaining tablets containing floating pellets with verapamil hydrochloride as API was examined. Mean production speed of tablet press was: single punch: 90–100 tablets/min; rotary: 500–600 tablets/min (25–30 rpm, 20 punches). On the basis of API release profiles from pellets before and after compression, the level of pellets shell damage was determined. Each formulation was characterized by conditional attributes describing: composition of tablet mass, tablet properties, type of the tablet press machine, compression force and the type of pellets polymer coating. Decision attribute classifying objects was similarity factor f_2_ of API release profiles from pellets before and after tableting. The determination of the release rate of API from pellet and tablet formulations was performed using the Ph. Eur. paddle apparatus with agitation speed of 75 rpm. Test were performed in 750 mL of hydrochloric acid (0.1 mol/L) at a temperature of 37 ± 0.5 °C. The dissolution time points for both profiles were the same: six time points, from 1 to 6 h. Table 1 presents condition attributes used in the study.

f_2_ was used for determining class of tablets since it constitute a relatively simple and widely accepted measure for comparing dissolution profiles. Regulatory authorities such as EMA [15] point the use of the f_2_ for this purpose, define requirements for its calculation (e.g., limitations for dissolution tests parameters) and its threshold value that prove the similarity. f_2_ factor was calculated using DDSolver, an extension to MS Excel, where the reference formulation was pellets coated with a polymer before compression, and the test formulation was pellets after compression (Equation (1)). This is one of the independent mathematical models allowing the comparison of release profiles [16].

(1) $$f_{2}=50\times \log\left[\frac{100}{\sqrt{1+\frac{\sum_{t=1}^{n}{\left(R\left(t\right)-T\left(t\right)\right)}^{2}}{n}}}\right]$$

where R and T are the percentage dissolved of the reference and test profile, respectively, at time point t; n is the number of sampling points.

In the course of analysis f_2_ values were discretized as follows:

- Tablets with API release profile similar to pellets—f_2_ ≥ 50—class 1
- Tablets with API release profile different from pellets—f_2_ < 50—class 2

### 2.2. Information System

The data set analysed using DRSA is organized as an information system in a tabular form, where a set of objects (formulations of tablets with pellets) is described by a finite set of condition attributes and one decision attribute (f_2_ similarity factor). Rows of such a table correspond to objects and columns to attributes, and at the intersection of rows and columns there are values called descriptors.

Table 2 presents a part of the information system describing a set of different formulations of tablets with pellets. The whole information system was built of 180 formulations and can be found in Supplementary Materials. 

### 2.3. Knowledge Discovery Technique

Data that concern technological parameters of solid dosage forms manufacturing can be seen as classification data. Classification concerns objects (formulations), described by condition attributes and a decision attribute, forming a decision table. Tableting process parameters and composition of tablets with pellets are condition attributes (independent variables). Class labels: 1 and 2 are assigned to formulations by a decision attribute (dependent variable). To explain the class assignment in terms of condition attributes, the rough set concept and its particular extension called dominance-based rough set approach (DRSA), was used. In the classical rough-set approach it is necessary to perform discretization of numerical scales of quantitative condition attributes, which is an invasive transformation of original data. In the case of DRSA, numerical attributes do not need to be discretized. DRSA proved to be an effective tool in analysis of classification data which are partially inconsistent. In the context of this work, inconsistency means that two formulations have similar description by condition attributes, while they are assigned to different similarity factor class. The rough sets representing classes discern between consistent and inconsistent formulations and prepare the ground for induction of decision rules from classification data structured in this way. DRSA assumes that the value sets (scales) of condition attributes are ordered and monotonically dependent on the order of decision classes. In consequence, the rules induced by DRSA are monotonic.

When a condition attribute is numerical and its value set is ordered such that the greater the value, the more likely is that the compound belongs to a better class, such attribute is called gain-type, and we say it is positively semantically correlated with the order of classes; analogously, when the smaller the value, the more likely is that a compound belongs to a better class, such attribute is called cost-type, and we say it is negatively semantically correlated with the order of classes. In elementary conditions of decision rules, gain-type attributes and cost-type attributes have opposite relation signs. In case of the type of data that we analyze, it is impossible to know a priori if attributes are gain- or cost-type, thus we are considering each original attribute in two copies, and for the first copy we assume it is gain-type, while for the second copy we assume it is cost-type. The applied transformation of data is non-invasive, i.e., it does not bias the matter of discovered relationships between condition attributes and the decision attribute. Then, the induction algorithm constructs decision rules involving elementary conditions on one or both copies of particular attributes.

In the course of the analysis, jRS and jMAF software based on DRSA methodology were used [17]. DRSA was chosen as the most suitable method to discover synthetic rules that exhibit monotonic relationships between tableting process parameters and composition of tablets with pellets, and their API release profiles.

### 2.4. Decision Rules

Decision rules represent the most important cause–effect relationships between values of condition attributes and the class assignment. A decision rule induced from an information table is denoted as *E*→*H*, which reads as “*if E, then H*”. A rule consists of a condition part (called also premise or evidence) *E*, and a conclusion (called also decision part, or prediction, or hypothesis) *H*. Considering a finite set of condition attributes *C* = {*q*_1_, *q*_2_, …, *q_n_*}, the condition part of the rule is a conjunction of elementary conditions on a particular subset of attributes:

*E* = *ei*_1_ ∧ *ei*_2_ ∧ … ∧ *ei_p_*,

where {*i*_1_, *i*_2_, …, *i_p_*} ⊆ {1, 2, …, *n*}, *p* ≤ *n*, and *ei_h_* is an elementary condition defined on the value set of attribute *qi_h_*, *h* ∈ {*i*_1_, *i*_2_, …, *i_p_*}, e.g., *ei_h_* ≡ *qi_h_* > 0.5, or *ei_h_* ≡ *qi_h_* = 1, or *ei_h_* ≡ *qi_h_* ∈ [0.5, 1]).

### 2.5. Bayesian Confirmation Measures

Attribute relevance measures that satisfy the property of Bayesian confirmation were considered [18]. These measures take into account interactions between attributes present in the decision rules. In this case, the property of confirmation is related to quantification of the degree to which the presence of an attribute in the premise of a rule provides evidence for or against the conclusion of the rule. The measure increases when more rules involving a given attribute suggest a correct decision, or when more rules that do not involve this attribute suggest an incorrect decision, otherwise it decreases [19]. The attributes with the highest values of the confirmation measure are the most relevant from the viewpoint of correct class assignment of a new product to the f_2_ decision class.

### 2.6. Stratified Cross-Validation

Since the SAR table, which is the basis of this analysis, consists of only 180 formulations, we used a 5-fold stratified cross-validation procedure to assess the predictive accuracy of rules. The cross-validation was repeated 100 times to obtain better reproducibility of results. Variable consistency bagging technique (VC-bagging) [20] was applied to generate sets of rules used in the further analysis. The predictive accuracy of DRSA decision rules obtained in this way was high (please see results section for more details). It was sufficient to consider these rules as useful guidelines for planning new formulations of MUPS. The generated sets of rules were further analysed to assess the relevance of the condition attributes.

## 3. Results

### 3.1. Decision Rules

Table 3 and Table 4 present two sets of 20 strongest decision rules obtained for class 1 tablets with pellets that maintain the release profiles of verapamil hydrochloride from pre-tableting and class 2 tablets that differ from the release profiles of non-tableted pellets, respectively. The rules presented are ranked according to the value of the Bayesian confirmation measure. Condition attributes that were not included in decision rules during the analysis were removed from the tables. The attribute “coating” has been divided into three attributes, one for each type of coating. For these attributes “0” means absence of that kind of coating, and “1” means presence. The decision rules contain the most important information characterizing the objects in class 1 and 2, that is, they show features leading to similar verapamil hydrochloride release profiles from pellets before and after tableting (f_2_ ≥ 50) and features leading to change in API release profiles after tableting (f_2_ < 50).

Strong decision rules, supported by a large number of objects, with a high confirmation measure, generated for class 1, allow to indicate condition attributes and their values leading to tableted pellets with API release profiles similar to pellet release profiles before tableting. Such rules are:

- Rule 1: If absence of ethyl cellulose coating and tablet mass ≤ 553.1 mg and crushing strength ≥ 68.8 (10^4^ N/m^2^) and amount of Avicel 102 ≥ 12.7%, then class 1;
- Rule 2: If presence of Eudragit NE coating and compression force > 6 kN and tablet mass ≤ 563.3 mg and hardness ≥ 42.4 N and absence of Avicel 101, then class 1;
- Rule 3: If presence of Eudragit NE coating and compression force > 6 kN and hardness ≥ 49.2 N and absence of Vivapur 200, Avicel 101, Povidone K30, then class 1;
- Rule 4: If presence of Eudragit NE coating and compression force > 6 kN and hardness ≥ 42.4 N and absence of Povidone K30 and Mannitol, then class 1;
- Rule 5: If presence of Eudragit NE coating and compression force > 6 kN and crushing strength ≥ 102.5 (10^4^ N/m^2^) and absence of Vivapur 200 and StarLac, then class 1.

Strong decision rules were also obtained for class 2 of tablets with pellets (Table 4). They provide information about attributes and their values that adversely affect the structure of the tableted pellets, i.e., the composition and parameters of the process, which are not worth using. Such rules are:

- Rule 1: If rotary tablet press and crushing strength ≤ 97.8 [10^4^ N/m^2^], then class 2;
- Rule 2: If absence of Eudragit NE coating and compression force < 18 kN and crushing strength ≤ 158.3 [10^4^ N/m^2^], then class 2;
- Rule 3: If absence of Eudragit NE coating and compression force < 18 kN and absence of Tablettose 80, then class 2;
- Rule 6: If crushing strength ≤ 68.1 [10^4^ N/m^2^] and Kollidon Cl ≥ 9.5%, then class 2;
- Rule 8: If compression force = 6 kN and crushing strength ≤ 72 [10^4^ N/m^2^], then class 2;

The obtained decision rules can be used as a guide to optimize the composition of pellets and tablet mass, as well as the parameters of the tableting process, leading to the preparation of tablets with a proper API release profile.

### 3.2. Predictive Attribute Revelance

**Figure 1:**
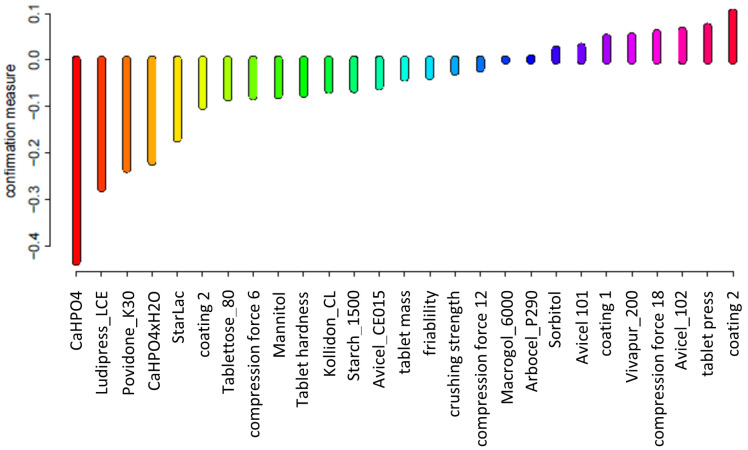
Predictive attribute relevance.

The results of the attribute relevance assessment for the generated sets of decision rules are presented in Figure 1. The higher the value of the confirmation measure of a conditional attribute, the greater its influence on the correct classification of objects using the generated rules. Attributes describing the composition (coating 2, coating 1, Avicel 101 and 102, Vivapur200) and the process (type of tablet press and compression force) have the greatest impact on the correct classification of objects.

### 3.3. Results of Stratified Cross-Validation

The average accuracy of the prediction is characterized by the results presented in Table 5. The same type of stratified cross-validation was performed with Random Forest and logistic regression to compare the results obtained with VC-bagging. Algorithms were implemented in WEKA toolkit [21]. Results show that VC-bagging is providing slightly better outcome than logistic regression and comparable to random forest, which is considered as an off-the-shelf robust classifier allowing to obtain very good predictive accuracy.

## 4. Discussion

Pellets are usually filled into capsules, but they can also form MUPS through compression to tablet after addition of excipients. As distinct from capsules, tablets are more convenient for patients, they can be divided into equal halves and their manufacturing process is more efficient and less expensive. However, the benefits of using MUPS do not affect the frequency of its use. This is mainly due to limited number of commercial MUPS available caused by the technological difficulties encountered during pellets tableting process [22].

In MUPS manufacturing there are several technological problems related to the mass uniformity, tablet hardness and friability. As can be seen in our study, choice of appropriate coating, type of tablet press, compression force, type and amount of excipients used, might be of special importance. However, the main technological challenge is to preserve the planned modification of API release from compressed pellets. It can be achieved by maintaining the structure of the pellet coating and its function to modify the release. Comparison of API release profiles from pellets before and after compression allows to determine the effect of the tableting process parameters and the composition of the tablet mass on the structure and properties of pellets. After oral administration, tablets with pellets should disintegrate rapidly, releasing individual pellets. Disintegration speed can affect the modification of the release profile in the initial minutes. During tableting, the pellets may clump, which also results in slower disintegration and affect the release of API [4]. Therefore, the choice of excipients and technological process parameters correlating with proper API release profiles require performing many experiments which are time consuming and associated with high costs. Application of machine learning methods such as DRSA, and its results provided as decision rules, can be helpful in significant reduction of the time and costs of formulation development [23].

The composition and thickness of the coating are the factors conditioning the release. If the coating is rigid and brittle, the polymer layer is immediately broken on the surface of the pellets. The plasticity of coating can be changed by the addition of plasticizers, e.g., propylene glycol (PG), triethyl citrate (TEC) [24]. Another factor affecting the API release profiles from MUPS after compression is the composition of pellets. Both the core and the coating should be flexible enough to change the shape under the influence of pressure. During compression, pellets should not crumble and break. The most commonly used polymers for coating that modifies the release of pellets are derivatives of acrylic acid (Eudragit^®^) and cellulose derivatives, mainly ethyl cellulose (Aquacoat^®^, Surelease^®^). The use of polyvinyl acetate (Kollicoat^®^ SR) and shellac is also described [22].

In our analysis, most decision rules describing formulations from class 1 (i.e., tablets with API release profile similar to pellets) determine the type of coating polymer. The attribute “coating 1—1” indicates the Eudragit NE film leading to the proper quality of tablets. At the same time, the attribute “coating 2—0” excludes the usefulness of EC. On the other hand, the decision rules describing class 2 (i.e., tablets with API release profile different from pellets, that is formulations in which the pellet coating could be damaged during tableting process) exclude Eudragit NE (“coating 1—0”) and include EC (“coating 2—1”). It indicates that the EC coated pellets form tablets with API release profile different from pellets itself. The discovered relationships are consistent with the observations reported in the literature related to the properties of used polymers [1,25]. Ethyl cellulose does not exhibit mechanical properties allowing the compression of particles coated with this polymer. EC is weak and brittle (elongation < 2%) and its films are not strengthened by addition of plasticizers [1]. Dashevsky et al. compared the release profiles from pellets with EC coating (Aquacoat ECD 30) before and after compression. 25% TEC additive did not improve elasticity of the coating enough to preserve it from damaging during tableting. The increase in the amount of API released as the compression force increased was also observed. Acrylic acid derivatives exhibit better mechanical properties that EC, therefore they are more suitable for coating pellets for MUPS manufacturing. Elongation of the Eudragit films (more than 75% for Eudragit RL/RS) allows tableting of pellets, without damaging the coating [25]. The presence of the “coating 1” attribute in most decision rules in class 1 confirms its usefulness in coating pellets for compression. This is mainly due to the elasticity of Eudragit NE.

Shellac is used in pharmaceutical technology as an enteric coating polymer due to its solubility at pH >7. It also shows good protective properties (low permeability of water and oxygen) and low toxicity (polymer approved as food additive) [26]. Shellac, however, is seldom used in the modified-release drug formulations. It is a natural polymer with a complex composition and the product series can vary quantitatively and/or qualitatively. In addition, changes in the structure and properties of the polymer can occur during storage, i.e., loss of enteric coating properties or modification in the film solubility [27]. The decision rules did not directly indicate the negative or positive effect of the shellac coating on the release of verapamil from pellets after tableting. However, rules defining lack of EC in objects from class 1 (coating 2—0) may indicate shellac as a polymer suitable for coating pellets.

Pellets, after mixing with the tablet mass, should be easily compressed under the minimum pressure, giving the tablet with proper hardness and low friability. The composition of the tablet mass affects the damage of the tableted pellets coating. Some excipients can protect the pellets coating during tableting. Torrado et al. noted the beneficial effect of the mixture of polyethylene glycol 3350, microcrystalline cellulose (MCC) and crospovidone (PVPP) on the integrity of the compressed pellets coating consisted of Eudragit RS with the addition of triacetin as a plasticizer. The protective properties were not exhibited by CaHPO_4_ [28]. One of the methods of improving the physical properties of pellets is the granulation of pellets with excipients. Pan et al. compressed pellets coated with Eudragit RS and L, which were granulated with MCC and PVPP. The addition of these substances protected the coating against damage [29]. Results of these studies demonstrate that MCC can act as a excipient protecting pellets coating from damaging in tableting process. Some of the rules describing objects from class 1 indicate one of the MCC pharmaceutical grades, Avicel^®^ 102 (≥12.7%), as a substance positively affecting the quality of tablets with pellets. Moreover, there are rules showing that absence of different MCC grade, Avicel^®^ 101, also leads to better release profile. Both grades of MCC show no significant difference in the compressibility. However, larger particle size of Avicel 102, being partially agglomerated product, generally provide better flow properties required for successful direct tableting [30].

In the decision rules, the “compression force” and “tablet press”, were important attributes affecting the correct classification of objects. However, the results are ambiguous. Class 1 is dominated by objects (tablets) obtained with a compression force >6 kN (12 or 18 kN). On the other hand, in class 2 there are objects obtained using the <18 kN compression force (6 or 12 kN). Thereby, proper quality tablets can be manufactured using a compression force higher than 6–12 kN. Scientific literature also reports ambiguous conclusions regarding the impact of the compression force on the API release profiles from compressed pellets. In the Bekert et al. study, no compression force effect was observed on the rate of API release from pellets [31]. However, in other studies, it was noticed that the higher compression force slows the release rate of API from pellets, while the lower force speeds it up. The influence of porosity and pellet size on the coating damage during tableting was also noted [12]. The attribute “tablet press—2” indicates that the rotary tablet press occurred mainly in the class 2 decision rules, together with an absence of Eudragit NE coating, low hardness and crushing strength attributes (force needed to crash a tablet, divided by multiplication of tablet radius and thickness). In class 1 there is one rule that indicates a usage of rotary tablet press with tablet hardness ≥42.9 N and presence of Eudragit NE coating. The conclusion is that it is worth using rotary tablet press only if the hardness of produced tablets is properly high (i.e., by using higher compression force) and the pellets are protected with elastic Eudragit NE. On the other hand, there is no rule that indicates usage of a single punch tablet press. It is related with differences in a compression mechanism. In a single punch tablet press the upper punch exerts pressure on a tablet mass, the lower punch only pushes the tablet from the die. The pressure during the tableting is asymmetrical, which may cause deformations in pellets coating, whereas, in the rotary tablet press there is a symmetrical pressure caused by both upper and lower punch. In addition, the pre-compression phase affects proper arrangement and uniformity of tablet mass in a die [12].

Tablet hardness has a significant impact on the implementation of final technological processes. Low hardness causes difficulties in coating, packaging or application, resulting in discomfort during swallowing. As it is apparent from the decision rules of class 1, tablets with a hardness higher than 42 N (up to >142 N) exhibited proper release profile. El Mahdi et al. listed factors affecting the hardness of tablets with pellets: compression force (increase results in a corresponding increase of hardness), type of excipients (depending on the mechanism of compression and the size of their particles) and percentage of excipients for pellets (the higher the percentage is, the higher the hardness of tablets) [32].

Machine learning methods are recently widely used for pharmaceutical formulation design and optimization. Artificial neural networks (ANN) are among the most popular techniques providing reliable results [23]. Our study provides a novel insight and propose DRSA as an alternative method for development of formulations and prediction of parameters associated with quality measures. DRSA explains classification decisions using intelligible decision rules that may be easily understood as scenarios of cause–effect relationships. The first application of DRSA in pharmaceutical technology was conducted by Pałkowski et al. [33]. DRSA has been applied to evaluate critical process parameters in manufacturing of pellets. This tool allowed to induce decision rules, along with Bayesian confirmation measures, which defined the important parameters influencing the quality of the obtained drug dosage forms. It was found that in obtaining spherical pellets the amount of water in the pellet mass, and the composition of the pellets (excipients used) were of the most important influence, taking into account considered technological parameters (spheronization time, speed, temperature, screw speed, and number of die holes).

## 5. Conclusions

DRSA appeared to be a useful method for discovering synthetic and intelligible decision rules that exhibit monotonic relationships between tableting process, composition of tablets with pellets, and their API release profiles. It can provide easy to interpret guidelines concerning parameters that are worth or not worth using in practice. Decision rules indicated that usage of specified compression force, coating and excipients leads to obtaining tablets with similar release profiles of verapamil hydrochloride in comparison to pellets from pre-tableting. These rules represent knowledge that can be used to optimize the manufacturing process of tablets with pellets.

## Figures and Tables

**Table 1:** Condition attributes and their domains.

Condition Attribute	Domain
Type of coating	1—Eudragit NE, 2—ethyl cellulose, 3—shellac
Tablet press	1—single punch, 2—rotary
Compression force (kN)	6, 12, 18
Tablet mass (mg)	516.0–568.0
Tablet hardness (N)	11.0–297.0
Crushing strength (10^4^ N/m^2^)	11.0–619.0
Friability (%)	0.00–10.1
Avicel PH 101 (%)	0, 12.7
Avicel PH 102 (%)	0, 12.7, 47.0
Mannitol (%)	0, 34.3
Tablettose 80 (%)	0, 34.3
Ludipress_LCE (%)	0, 34.3
Sorbitol (%)	0, 34.3
Povidone_K30 (%)	0, 34.3
StarLac (%)	0, 34.3
CaHPO_4_xH_2_O (%)	0, 34.3
CaHPO_4_ (%)	0, 34.3
Vivapur_200 (%)	0, 34.3
Avicel_CE015 (%)	0, 34.3
Macrogol_6000 (%)	0, 34.3
Starch_1500 (%)	0, 43.8
Arbocel_P290 (%)	0, 47
Kollidon_CL (%)	0, 9.5

**Table 2:** A part of the information system.

No. of Formulation	Coating	Tablet Press	Compression Force (kN)	Tablet Mass (mg)	Tablet Hardness (N)	Crushing Strength (10^4^ N/m^2^)	Friability (%)	Avicel_102 (%)	Avicel_101 (%)	Mannitol (%)	Tablettose_80 (%)	Ludipress_LCE (%)	Arbocel_P290 (%)	Sorbitol (%)	Povidone_K30 (%)	StarLac (%)	Starch_1500 (%)	CaHPO4xH2O (%)	CaHPO4 (%)	Vivapur_200 (%)	Avicel_CE015 (%)	Macrogol_6000 (%)	Kollidon_CL (%)	f_2_
1	1	1	6	559	63	92	2.30	12.7	0	34.3	0	0	0	0	0	0	0	0	0	0	0	0	9.5	59.4
2	1	1	6	548	66	96	3.10	0	12.7	34.3	0	0	0	0	0	0	0	0	0	0	0	0	9.5	56.3
3	1	1	6	557	111	163	4.20	0	12.7	0	0	0	0	0	0	0	0	0	0	0	34.3	0	9.5	35.7
4	1	1	6	558	96	140	0.50	47	0	0	0	0	0	0	0	0	0	0	0	0	0	0	9.5	37.5
5	1	1	6	562	41	58	0.00	12.7	0	0	34.3	0	0	0	0	0	0	0	0	0	0	0	9.5	38.7
6	1	1	6	557	45	67	2.40	12.7	0	0	0	34.3	0	0	0	0	0	0	0	0	0	0	9.5	35.9
7	1	1	6	562	105	153	0.60	0	0	0	0	0	47	0	0	0	0	0	0	0	0	0	9.5	40.9
8	1	1	6	559	76	111	0.40	12.7	0	0	0	0	0	34.3	0	0	0	0	0	0	0	0	9.5	59.4
9	1	1	6	555	55	80	0.70	12.7	0	0	0	0	0	0	34.3	0	0	0	0	0	0	0	9.5	56.3
10	1	1	6	565	36	52	2.30	12.7	0	0	0	0	0	0	0	34.3	0	0	0	0	0	0	9.5	35.7
11	1	1	6	566	26	38	3.40	12.7	0	0	0	0	0	0	0	0	43.8	0	0	0	0	0	0	37.5
12	1	1	6	559	40	58	1.20	12.7	0	0	0	0	0	0	0	0	0	34.3	0	0	0	0	9.5	38.7
13	1	1	6	560	39	57	0.90	12.7	0	0	0	0	0	0	0	0	0	0	34.3	0	0	0	9.5	35.9
14	1	1	6	562	40	59	0.70	12.7	0	0	0	0	0	0	0	0	0	0	0	34.3	0	0	9.5	40.9
15	1	1	6	550	40	58	0.40	12.7	0	0	0	0	0	0	0	0	0	0	0	0	34.3	0	9.5	59.4
16	1	1	6	555	40	58	0.60	12.7	0	0	0	0	0	0	0	0	0	0	0	0	0	34.3	9.5	55.9
17	1	1	12	549	91	134	1.10	12.7	0	34.3	0	0	0	0	0	0	0	0	0	0	0	0	9.5	50.7
18	1	1	12	545	79	115	0.90	0	12.7	34.3	0	0	0	0	0	0	0	0	0	0	0	0	9.5	63.5
19	1	1	12	554	248	363	0.90	0	12.7	0	0	0	0	0	0	0	0	0	0	0	34.3	0	9.5	48.3
20	1	1	12	566	229	335	0.40	47	0	0	0	0	0	0	0	0	0	0	0	0	0	0	9.5	58.7

**Table 3:** Decision rules for class 1 pellets.

No. of Rule	Coating_1	Coating_2	Tablet Press	Compression Force (kN)	Tablet Mass (mg)	Hardness (N)	Crushing Strength (10^4^ N/m^2^)	Friability (%)	Avicel_102 (%)	Vivapur_200 (%)	Avicel_101 (%)	Povidone_K30 (%)	StarLac (%)	Tablettose_80 (%)	Mannitol (%)	Rule Support	Rule Strength	Confirmation Measure
1	-	0	-	-	≤553.1	-	≥68.8		≥12.7	-	-	-	-	-	-	32	0.1777	0.75
2	1	-	-	>6	≤563.3	≥42.4	-	-	-	-	0	-	-	-	-	46	0.2555	0.75
3	1	-	-	>6	-	≥49.2	-	-	-	0	0	0	-	-	-	43	0.2388	0.73
4	1	-	-	>6	-	≥42.4	-	-	-	-	-	0	-	-	0	47	0.2611	0.73
5	1	-	-	>6	-	-	≥102.5	-	-	0	-	-	0	-	-	48	0.2666	0.73
6	1	-	-	-	-	≥105.8	-	-	-	-	0	-	-	-	-	33	0.1833	0.72
7	1	-	-	>6	-	≥105	-	-	-	-	-	-	-	-	-	36	0.2000	0.72
8	-	-	-	-	-	>111.9	>163.6	-	-	-	-	-	-	-	-	36	0.2000	0.72
9	-	0	-	-	-	-	≥180.8	-	-	-	-	-	-	-	-	39	0.2166	0.72
10	-	0	-	-	-	≥107	-	-	-	-	-	-	-	-	-	41	0.2277	0.72
11	1	-	-	-	≤556.4	-	-	1.6	-	-	-	-	-	-	-	50	0.2777	0.72
12	1	-	-	-	≤554.1	-	-	1.1	-	-	-	-	-	-	-	34	0.1888	0.70
13	1	-	2	-	-	≥49.2	-	-	-	-	-	-	-	-	-	37	0.2055	0.70
14	1	-	-	>6	≤561.5	≥49.2	-	-	-	0	0	-	-	-	-	41	0.2277	0.70
15	1	-	-	>6	-	>42.4	-	-	-	-	-	0	-	-	-	53	0.2944	0.70
16	-	-	-	-	-	≥148.9	-	-	-	0	-	-	-	-	-	21	0.1166	0.69
17	1	-	-	-	≤554.1	-	-	-	≥12.7	-	-	-	-	-	-	32	0.1777	0.69
18	1	-	-	>6	-	-	-	-	-	0	0	0	0	-	-	43	0.2388	0.69
19	1	-	-	>6	-	≥49.2	-	-	-	0	-	-	0	-	0	43	0.2388	0.69
20	-	0	-	-	-	-	≥163.6	-	-	-	-	-	-	-	-	47	0.2611	0.69

**Table 4:** Decision rules for class 2 pellets.

No. of Rule	Coating_1	Coating_2	Tablet Press	Compression Force (kN)	Tablet Mass (mg)	Hardness (N)	Crushing Strength (10^4^ N/m^2^)	Avicel_CE015 (%)	Kollidon_Cl (%)	Tablettose_80 (%)	Macrogol 6000 (%)	Arbocel_P290 (%)	Rule Support	Rule Strength	Confirmation Measure
1	-	-	2	-	-	-	≤97.8	-	-	-	-	-	30	0.1666	0.70
2	0	-	-	<18	-	-	≤158.3	-	-	-	-	-	48	0.2666	0.70
3	0	-	-	<18	-	-	-	-	-	0	-	-	42	0.2333	0.70
4	0	-	-	<18	-	-	≤133.1	-	-	-	-	-	43	0.2388	0.70
5	0	-	2	-	-	-	-	-	-	-	-	0	31	0.1722	0.70
6	-	-	-	-	-	-	≤68.1	-	9.5	-	-	-	37	0.2055	0.69
7	0	-	-	<18	-	-	-	0	-	-	-	-	42	0.2333	0.67
8	-	-	-	6	-	-	≤72	-	-	-	-	-	31	0.1722	0.67
9	0	-	-	12	-	-	-	-	-	-	-	-	23	0.1277	0.67
10	-	-	2	-	-	≤48.8	-	-	-	-	-	-	23	0.1277	0.67
11	-	-	2	-	-	≤60.1	-	-	-	-	-	-	31	0.1722	0.67
12	0	-	-	<18	<558	-	-	-	-	-	-	-	41	0.2277	0.66
13	0	-	2	-	-	≤84.5	-	-	-	-	-	-	30	0.1666	0.66
14	0	-	-	<18	-	-	≤90.9	-	-	-	-	-	37	0.2055	0.66
15	-	-	-	<18	-	-	≤67.3	-	-	-	-	-	35	0.1944	0.66
16	-	1	-	-	-	-	-	-	-	-	-	-	42	0.2333	0.65
17	0	-	2	-	-	-	≤158.3	-	-	-	-	-	36	0.2000	0.65
18	0	-	2	-	-	-	≤138	-	-	-	-	-	33	0.1833	0.65
19	-	-	-	<18	-	-	≤68.1	-	-	-	-	-	36	0.2000	0.65
20	0	-	2	-	-	-	≤148.5	-	-	-	-	-	34	0.1888	0.65

**Table 5:** Cross-validation results.

	VC-Bagging		Random Forest		Logistic Regression	
	(Avg. No.)	(Avg. %)	(Avg. No.)	(Avg. %)	(Avg. No.)	(Avg. %)
Correctly Classified Instances	148.91	82.72	149.11	80.24	141.02	78.34
Incorrectly Classified Instances	31.09	17.27	30.89	19.76	38.98	21.65
Average Classification Accuracy [%]	82.72		82.74		78.32	
Average Precision [%]	82.75		83.12		78.40	

## Supplementary Materials

The following are available online at https://www.mdpi.com/1999-4923/12/11/1024/s1, Table S1: Information system.
