# Supplementary material for: Application of Dominance-Based Rough Set Approach for Optimization of Pellets Tableting Process
Source: Pharmaceutics. 2020 Oct 26;12(11):1024. doi: 10.3390/pharmaceutics12111024 (PMC7692369; doi:10.3390/pharmaceutics12111024)
Supplement: Supplementary file 1 [file pharmaceutics-12-01024-s001.pdf]

pharmaceutics

MDPI

Supplementary Materials

Application of Dominance-Based Rough Set Approach for Optimization of Pellets Tableting Process

Maciej Karolak, Łukasz Pałkowski, Bartłomiej Kubiak, Jerzy Błaszczyński, Rafał Łunio, Wiesław Sawicki, Roman Słowiński and Jerzy Krysiński

Table 1. Information system.

No. of formulation	Coating	Tablet press	Compression force [kN]	Tablet mass [mg]	Tablet hardness [N]	Crushing strength [10^4 N/m^2]	Friability [%]	Avicel_102 [%]	Avicel_101 [%]	Mannitol [%]	Tablettose_80 [%]	Ludipress_LCE [%]	Arbocel_P290 [%]	Sorbitol [%]	Povidone_K30 [%]	StarLac [%]	Starch_1500 [%]	CaHPO4xH2O [%]	CaHPO4 [%]	Vivapur_200 [%]	Avicel_CE015 [%]	Macrogol_6000 [%]	Kollidon_CL [%]	f_2
**1**	1	1	6	559	63	92	2.30	12.7	0	34.3	0	0	0	0	0	0	0	0	0	0	0	0	9.5	59.4
**2**	1	1	6	548	66	96	3.10	0	12.7	34.3	0	0	0	0	0	0	0	0	0	0	0	0	9.5	56.3
**3**	1	1	6	557	111	163	4.20	0	12.7	0	0	0	0	0	0	0	0	0	0	0	34.3	0	9.5	35.7
**4**	1	1	6	558	96	140	0.50	47	0	0	0	0	0	0	0	0	0	0	0	0	0	0	9.5	37.5
**5**	1	1	6	562	41	58	0.00	12.7	0	0	34.3	0	0	0	0	0	0	0	0	0	0	0	9.5	38.7
**6**	1	1	6	557	45	67	2.40	12.7	0	0	0	34.3	0	0	0	0	0	0	0	0	0	0	9.5	35.9
**7**	1	1	6	562	105	153	0.60	0	0	0	0	0	47	0	0	0	0	0	0	0	0	0	9.5	40.9
**8**	1	1	6	559	76	111	0.40	12.7	0	0	0	0	0	34.3	0	0	0	0	0	0	0	0	9.5	59.4
**9**	1	1	6	555	55	80	0.70	12.7	0	0	0	0	0	0	34.3	0	0	0	0	0	0	0	9.5	56.3
**10**	1	1	6	565	36	52	2.30	12.7	0	0	0	0	0	0	0	34.3	0	0	0	0	0	0	9.5	35.7
**11**	1	1	6	566	26	38	3.40	12.7	0	0	0	0	0	0	0	0	43.8	0	0	0	0	0	0	37.5
**12**	1	1	6	559	40	58	1.20	12.7	0	0	0	0	0	0	0	0	0	34.3	0	0	0	0	9.5	38.7
**13**	1	1	6	560	39	57	0.90	12.7	0	0	0	0	0	0	0	0	0	0	34.3	0	0	0	9.5	35.9
**14**	1	1	6	562	40	59	0.70	12.7	0	0	0	0	0	0	0	0	0	0	0	34.3	0	0	9.5	40.9
**15**	1	1	6	550	40	58	0.40	12.7	0	0	0	0	0	0	0	0	0	0	0	0	34.3	0	9.5	59.4

**16**	1	1	6	555	40	58	0.60	12.7	0	0	0	0	0	0	0	0	0	0	0	0	0	34.3	9.5	55.9
**17**	1	1	12	549	91	134	1.10	12.7	0	34.3	0	0	0	0	0	0	0	0	0	0	0	0	9.5	50.7
**18**	1	1	12	545	79	115	0.90	0	12.7	34.3	0	0	0	0	0	0	0	0	0	0	0	0	9.5	63.5
**19**	1	1	12	554	248	363	0.90	0	12.7	0	0	0	0	0	0	0	0	0	0	0	34.3	0	9.5	48.3
**20**	1	1	12	566	229	335	0.40	47	0	0	0	0	0	0	0	0	0	0	0	0	0	0	9.5	58.7
**21**	1	1	12	549	72	105	1.60	12.7	0	0	34.3	0	0	0	0	0	0	0	0	0	0	0	9.5	40.3
**22**	1	1	12	558	112	164	0.90	12.7	0	0	0	34.3	0	0	0	0	0	0	0	0	0	0	9.5	43.6
**23**	1	1	12	555	155	227	0.40	0	0	0	0	0	47	0	0	0	0	0	0	0	0	0	9.5	49.1
**24**	1	1	12	556	260	380	0.10	12.7	0	0	0	0	0	34.3	0	0	0	0	0	0	0	0	9.5	44.9
**25**	1	1	12	555	67	98	0.20	12.7	0	0	0	0	0	0	34.3	0	0	0	0	0	0	0	9.5	30.0
**26**	1	1	12	568	92	134	0.60	12.7	0	0	0	0	0	0	0	34.3	0	0	0	0	0	0	9.5	38.4
**27**	1	1	12	555	36	53	1.60	12.7	0	0	0	0	0	0	0	0	43.8	0	0	0	0	0	0	38.3
**28**	1	1	12	564	69	100	0.40	12.7	0	0	0	0	0	0	0	0	0	34.3	0	0	0	0	9.5	44.3
**29**	1	1	12	545	79	115	0.40	12.7	0	0	0	0	0	0	0	0	0	0	34.3	0	0	0	9.5	51.3
**30**	1	1	12	558	160	234	0.10	12.7	0	0	0	0	0	0	0	0	0	0	0	34.3	0	0	9.5	52.0
**31**	1	1	12	556	106	155	0.20	12.7	0	0	0	0	0	0	0	0	0	0	0	0	34.3	0	9.5	60.1
**32**	1	1	12	562	182	266	0.20	12.7	0	0	0	0	0	0	0	0	0	0	0	0	0	34.3	9.5	77.3
**33**	1	1	18	555	112	164	1.00	12.7	0	34.3	0	0	0	0	0	0	0	0	0	0	0	0	9.5	41.6
**34**	1	1	18	561	87	127	0.30	0	12.7	34.3	0	0	0	0	0	0	0	0	0	0	0	0	9.5	39.3
**35**	1	1	18	558	273	421	0.30	0	12.7	0	0	0	0	0	0	0	0	0	0	0	34.3	0	9.5	64.6
**36**	1	1	18	549	245	358	0.20	47	0	0	0	0	0	0	0	0	0	0	0	0	0	0	9.5	50.1
**37**	1	1	18	556	80	117	0.70	12.7	0	0	34.3	0	0	0	0	0	0	0	0	0	0	0	9.5	44.5
**38**	1	1	18	562	131	192	0.30	12.7	0	0	0	34.3	0	0	0	0	0	0	0	0	0	0	9.5	54.6
**39**	1	1	18	549	167	244	0.00	0	0	0	0	0	47	0	0	0	0	0	0	0	0	0	9.5	57.5
**40**	1	1	18	556	282	412	0.10	12.7	0	0	0	0	0	34.3	0	0	0	0	0	0	0	0	9.5	52.0
**41**	1	1	18	566	72	106	0.10	12.7	0	0	0	0	0	0	34.3	0	0	0	0	0	0	0	9.5	26.8
**42**	1	1	18	558	120	176	0.40	12.7	0	0	0	0	0	0	0	34.3	0	0	0	0	0	0	9.5	39.2
**43**	1	1	18	564	42	62	0.20	12.7	0	0	0	0	0	0	0	0	43.8	0	0	0	0	0	0	48.4
**44**	1	1	18	554	89	130	0.20	12.7	0	0	0	0	0	0	0	0	0	34.3	0	0	0	0	9.5	46.9
**45**	1	1	18	550	107	156	0.20	12.7	0	0	0	0	0	0	0	0	0	0	34.3	0	0	0	9.5	60.5
**46**	1	1	18	555	264	386	0.10	12.7	0	0	0	0	0	0	0	0	0	0	0	34.3	0	0	9.5	50.8
**47**	1	1	18	549	141	206	0.10	12.7	0	0	0	0	0	0	0	0	0	0	0	0	34.3	0	9.5	49.8

**48**	1	1	18	557	208	304	0.10	12.7	0	0	0	0	0	0	0	0	0	0	0	0	0	34.3	9.5	54.0
**49**	1	2	6	546	63	105	1.60	12.7	0	34.3	0	0	0	0	0	0	0	0	0	0	0	0	9.5	47.4
**50**	1	2	6	549	71	119	1.60	0	12.7	34.3	0	0	0	0	0	0	0	0	0	0	0	0	9.5	55.0
**51**	1	2	6	548	127	212	3.50	0	12.7	0	0	0	0	0	0	0	0	0	0	0	34.3	0	9.5	34.1
**52**	1	2	6	559	102	171	0.10	47	0	0	0	0	0	0	0	0	0	0	0	0	0	0	9.5	36.0
**53**	1	2	6	553	57	95	1.80	12.7	0	0	34.3	0	0	0	0	0	0	0	0	0	0	0	9.5	37.5
**54**	1	2	6	561	68	113	1.00	12.7	0	0	0	34.3	0	0	0	0	0	0	0	0	0	0	9.5	34.1
**55**	1	2	6	549	102	169	0.50	0	0	0	0	0	47	0	0	0	0	0	0	0	0	0	9.5	40.2
**56**	1	2	6	556	127	212	0.30	12.7	0	0	0	0	0	34.3	0	0	0	0	0	0	0	0	9.5	51.0
**57**	1	2	6	554	63	105	0.60	12.7	0	0	0	0	0	0	34.3	0	0	0	0	0	0	0	9.5	49.9
**58**	1	2	6	545	47	78	1.60	12.7	0	0	0	0	0	0	0	34.3	0	0	0	0	0	0	9.5	40.7
**59**	1	2	6	566	23	38	1.80	12.7	0	0	0	0	0	0	0	0	43.8	0	0	0	0	0	0	35.8
**60**	1	2	6	557	40	67	1.00	12.7	0	0	0	0	0	0	0	0	0	34.3	0	0	0	0	9.5	37.8
**61**	1	2	6	557	36	60	0.80	12.7	0	0	0	0	0	0	0	0	0	0	34.3	0	0	0	9.5	39.8
**62**	1	2	6	552	68	113	0.60	12.7	0	0	0	0	0	0	0	0	0	0	0	34.3	0	0	9.5	44.1
**63**	1	2	6	540	51	85	0.30	12.7	0	0	0	0	0	0	0	0	0	0	0	0	34.3	0	9.5	49.6
**64**	1	2	6	551	62	104	0.50	12.7	0	0	0	0	0	0	0	0	0	0	0	0	0	34.3	9.5	61.0
**65**	1	2	12	549	92	191	0.60	12.7	0	34.3	0	0	0	0	0	0	0	0	0	0	0	0	9.5	51.7
**66**	1	2	12	544	81	169	0.80	0	12.7	34.3	0	0	0	0	0	0	0	0	0	0	0	0	9.5	53.1
**67**	1	2	12	561	223	464	0.80	0	12.7	0	0	0	0	0	0	0	0	0	0	0	34.3	0	9.5	52.9
**68**	1	2	12	546	211	439	0.30	47	0	0	0	0	0	0	0	0	0	0	0	0	0	0	9.5	57.1
**69**	1	2	12	548	82	171	1.30	12.7	0	0	34.3	0	0	0	0	0	0	0	0	0	0	0	9.5	53.9
**70**	1	2	12	548	127	264	0.80	12.7	0	0	0	34.3	0	0	0	0	0	0	0	0	0	0	9.5	41.8
**71**	1	2	12	565	149	310	0.30	0	0	0	0	0	47	0	0	0	0	0	0	0	0	0	9.5	47.7
**72**	1	2	12	546	276	575	0.10	12.7	0	0	0	0	0	34.3	0	0	0	0	0	0	0	0	9.5	53.9
**73**	1	2	12	554	67	140	0.10	12.7	0	0	0	0	0	0	34.3	0	0	0	0	0	0	0	9.5	56.0
**74**	1	2	12	546	73	152	0.50	12.7	0	0	0	0	0	0	0	34.3	0	0	0	0	0	0	9.5	54.8
**75**	1	2	12	552	33	68	1.30	12.7	0	0	0	0	0	0	0	0	43.8	0	0	0	0	0	0	43.0
**76**	1	2	12	562	73	151	0.30	12.7	0	0	0	0	0	0	0	0	0	34.3	0	0	0	0	9.5	55.0
**77**	1	2	12	544	82	170	0.30	12.7	0	0	0	0	0	0	0	0	0	0	34.3	0	0	0	9.5	58.1
**78**	1	2	12	559	162	337	0.10	12.7	0	0	0	0	0	0	0	0	0	0	0	34.3	0	0	9.5	49.4
**79**	1	2	12	546	117	243	0.10	12.7	0	0	0	0	0	0	0	0	0	0	0	0	34.3	0	9.5	58.4

**80**	1	2	12	541	193	403	0.10	12.7	0	0	0	0	0	0	0	0	0	0	0	0	0	34.3	9.5	65.7
**81**	1	2	18	552	121	251	0.80	12.7	0	34.3	0	0	0	0	0	0	0	0	0	0	0	0	9.5	41.9
**82**	1	2	18	544	94	195	0.10	0	12.7	34.3	0	0	0	0	0	0	0	0	0	0	0	0	9.5	37.2
**83**	1	2	18	548	269	560	0.10	0	12.7	0	0	0	0	0	0	0	0	0	0	0	34.3	0	9.5	52.6
**84**	1	2	18	540	257	535	0.10	47	0	0	0	0	0	0	0	0	0	0	0	0	0	0	9.5	45.3
**85**	1	2	18	551	93	193	0.60	12.7	0	0	34.3	0	0	0	0	0	0	0	0	0	0	0	9.5	70.9
**86**	1	2	18	554	139	290	0.10	12.7	0	0	0	34.3	0	0	0	0	0	0	0	0	0	0	9.5	53.3
**87**	1	2	18	546	173	360	0.00	0	0	0	0	0	47	0	0	0	0	0	0	0	0	0	9.5	55.0
**88**	1	2	18	553	297	619	0.10	12.7	0	0	0	0	0	34.3	0	0	0	0	0	0	0	0	9.5	67.3
**89**	1	2	18	534	81	169	0.10	12.7	0	0	0	0	0	0	34.3	0	0	0	0	0	0	0	9.5	51.3
**90**	1	2	18	559	110	229	0.30	12.7	0	0	0	0	0	0	0	34.3	0	0	0	0	0	0	9.5	54.8
**91**	1	2	18	562	49	103	0.10	12.7	0	0	0	0	0	0	0	0	43.8	0	0	0	0	0	0	48.5
**92**	1	2	18	552	93	194	0.10	12.7	0	0	0	0	0	0	0	0	0	34.3	0	0	0	0	9.5	49.0
**93**	1	2	18	557	112	233	0.10	12.7	0	0	0	0	0	0	0	0	0	0	34.3	0	0	0	9.5	51.7
**94**	1	2	18	562	262	546	0.10	12.7	0	0	0	0	0	0	0	0	0	0	0	34.3	0	0	9.5	38.6
**95**	1	2	18	559	168	350	0.10	12.7	0	0	0	0	0	0	0	0	0	0	0	0	34.3	0	9.5	55.0
**96**	1	2	18	559	274	571	0.10	12.7	0	0	0	0	0	0	0	0	0	0	0	0	0	34.3	9.5	67.4
**97**	2	1	6	551	17	47	0.00	12.7	0	34.3	0	0	0	0	0	0	0	0	0	0	0	0	9.5	25.9
**98**	2	1	6	550	19	31	0.00	0	12.7	34.3	0	0	0	0	0	0	0	0	0	0	0	0	9.5	23.5
**99**	2	1	6	552	41	31	0.00	0	12.7	0	0	0	0	0	0	0	0	0	0	0	34.3	0	9.5	20.0
**100**	2	1	6	555	13	36	0.00	12.7	0	0	34.3	0	0	0	0	0	0	0	0	0	0	0	9.5	21.1
**101**	2	1	6	553	31	51	1.80	0	0	0	0	0	47	0	0	0	0	0	0	0	0	0	9.5	20.1
**102**	2	1	6	554	37	60	0.36	12.7	0	0	0	0	0	34.3	0	0	0	0	0	0	0	0	9.5	20.0
**103**	2	1	6	560	62	103	0.10	12.7	0	0	0	0	0	0	0	0	0	0	0	0	0	34.3	9.5	18.9
**104**	2	1	12	561	41	69	1.30	12.7	0	34.3	0	0	0	0	0	0	0	0	0	0	0	0	9.5	26.1
**105**	2	1	12	541	36	63	0.50	0	12.7	34.3	0	0	0	0	0	0	0	0	0	0	0	0	9.5	30.6
**106**	2	1	12	554	52	87	0.30	0	12.7	0	0	0	0	0	0	0	0	0	0	0	34.3	0	9.5	17.3
**107**	2	1	12	558	47	86	0.00	12.7	0	0	34.3	0	0	0	0	0	0	0	0	0	0	0	9.5	19.6
**108**	2	1	12	551	86	143	0.60	0	0	0	0	0	47	0	0	0	0	0	0	0	0	0	9.5	20.2
**109**	2	1	12	559	66	111	0.00	12.7	0	0	0	0	0	34.3	0	0	0	0	0	0	0	0	9.5	33.5
**110**	2	1	12	566	109	169	0.10	12.7	0	0	0	0	0	0	0	0	0	0	0	0	0	34.3	9.5	18.8
**111**	2	1	18	552	58	91	1.10	12.7	0	34.3	0	0	0	0	0	0	0	0	0	0	0	0	9.5	17.3

**112**	2	1	18	556	62	101	0.10	0	12.7	34.3	0	0	0	0	0	0	0	0	0	0	0	0	9.5	19.4
**113**	2	1	18	549	61	100	0.10	0	12.7	0	0	0	0	0	0	0	0	0	0	0	34.3	0	9.5	21.6
**114**	2	1	18	556	56	101	0.80	12.7	0	0	34.3	0	0	0	0	0	0	0	0	0	0	0	9.5	20.3
**115**	2	1	18	552	101	169	0.10	0	0	0	0	0	47	0	0	0	0	0	0	0	0	0	9.5	17.5
**116**	2	1	18	558	114	174	0.10	12.7	0	0	0	0	0	34.3	0	0	0	0	0	0	0	0	9.5	31.2
**117**	2	1	18	549	111	182	0.10	12.7	0	0	0	0	0	0	0	0	0	0	0	0	0	34.3	9.5	16.5
**118**	2	2	6	556	16	15	0.00	12.7	0	34.3	0	0	0	0	0	0	0	0	0	0	0	0	9.5	20.5
**119**	2	2	6	542	17	30	0.00	0	12.7	34.3	0	0	0	0	0	0	0	0	0	0	0	0	9.5	24.5
**120**	2	2	6	537	19	16	0.00	0	12.7	0	0	0	0	0	0	0	0	0	0	0	34.3	0	9.5	25.1
**121**	2	2	6	543	13	11	0.00	12.7	0	0	34.3	0	0	0	0	0	0	0	0	0	0	0	9.5	18.1
**122**	2	2	6	551	16	36	1.60	0	0	0	0	0	47	0	0	0	0	0	0	0	0	0	9.5	19.2
**123**	2	2	6	541	16	37	0.00	12.7	0	0	0	0	0	34.3	0	0	0	0	0	0	0	0	9.5	19.2
**124**	2	2	6	551	81	112	0.10	12.7	0	0	0	0	0	0	0	0	0	0	0	0	0	34.3	9.5	25.7
**125**	2	2	12	552	26	41	3.10	12.7	0	34.3	0	0	0	0	0	0	0	0	0	0	0	0	9.5	22.5
**126**	2	2	12	536	27	44	0.00	0	12.7	34.3	0	0	0	0	0	0	0	0	0	0	0	0	9.5	20.5
**127**	2	2	12	543	30	86	10.10	0	12.7	0	0	0	0	0	0	0	0	0	0	0	34.3	0	9.5	24.0
**128**	2	2	12	552	36	57	1.10	12.7	0	0	34.3	0	0	0	0	0	0	0	0	0	0	0	9.5	19.0
**129**	2	2	12	549	51	82	0.90	0	0	0	0	0	47	0	0	0	0	0	0	0	0	0	9.5	22.5
**130**	2	2	12	547	64	156	0.10	12.7	0	0	0	0	0	34.3	0	0	0	0	0	0	0	0	9.5	22.5
**131**	2	2	12	547	118	108	0.10	12.7	0	0	0	0	0	0	0	0	0	0	0	0	0	34.3	9.5	30.8
**132**	2	2	18	551	37	62	0.50	12.7	0	34.3	0	0	0	0	0	0	0	0	0	0	0	0	9.5	24.4
**133**	2	2	18	547	52	61	3.10	0	12.7	34.3	0	0	0	0	0	0	0	0	0	0	0	0	9.5	24.0
**134**	2	2	18	542	54	62	0.10	0	12.7	0	0	0	0	0	0	0	0	0	0	0	34.3	0	9.5	26.5
**135**	2	2	18	552	56	69	0.60	12.7	0	0	34.3	0	0	0	0	0	0	0	0	0	0	0	9.5	25.5
**136**	2	2	18	547	89	116	0.50	0	0	0	0	0	47	0	0	0	0	0	0	0	0	0	9.5	21.8
**137**	2	2	18	541	114	106	0.10	12.7	0	0	0	0	0	34.3	0	0	0	0	0	0	0	0	9.5	21.8
**138**	2	2	18	547	119	111	0.10	12.7	0	0	0	0	0	0	0	0	0	0	0	0	0	34.3	9.5	28.1
**139**	3	1	6	551	11	23	0.00	12.7	0	34.3	0	0	0	0	0	0	0	0	0	0	0	0	9.5	45.6
**140**	3	1	6	554	11	22	0.00	0	12.7	34.3	0	0	0	0	0	0	0	0	0	0	0	0	9.5	29.0
**141**	3	1	6	550	16	26	0.00	0	12.7	0	0	0	0	0	0	0	0	0	0	0	34.3	0	9.5	34.7
**142**	3	1	6	551	16	23	0.00	12.7	0	0	34.3	0	0	0	0	0	0	0	0	0	0	0	9.5	34.8
**143**	3	1	6	556	55	91	0.18	0	0	0	0	0	47	0	0	0	0	0	0	0	0	0	9.5	28.1

**144**	3	1	6	561	44	68	0.36	12.7	0	0	0	0	0	34	0	0	0	0	0	0	0	0	9.5	34.0
**145**	3	1	6	551	80	126	0.18	12.7	0	0	0	0	0	0	0	0	0	0	0	0	0	34.3	9.5	48.4
**146**	3	1	12	553	42	69	0.36	12.7	0	34.3	0	0	0	0	0	0	0	0	0	0	0	0	9.5	45.6
**147**	3	1	12	546	45	82	0.36	0	12.7	34.3	0	0	0	0	0	0	0	0	0	0	0	0	9.5	34.0
**148**	3	1	12	555	59	94	0.36	0	12.7	0	0	0	0	0	0	0	0	0	0	0	34.3	0	9.5	45.0
**149**	3	1	12	563	42	68	0.36	12.7	0	0	34.3	0	0	0	0	0	0	0	0	0	0	0	9.5	44.6
**150**	3	1	12	551	89	138	0.00	0	0	0	0	0	47	0	0	0	0	0	0	0	0	0	9.5	35.4
**151**	3	1	12	556	113	180	0.06	12.7	0	0	0	0	0	34.3	0	0	0	0	0	0	0	0	9.5	52.5
**152**	3	1	12	559	69	151	0.06	12.7	0	0	0	0	0	0	0	0	0	0	0	0	0	34.3	9.5	52.3
**153**	3	1	18	546	57	88	0.06	12.7	0	34.3	0	0	0	0	0	0	0	0	0	0	0	0	9.5	42.8
**154**	3	1	18	551	61	101	0.18	0	12.7	34.3	0	0	0	0	0	0	0	0	0	0	0	0	9.5	46.1
**155**	3	1	18	544	83	136	0.00	0	12.7	0	0	0	0	0	0	0	0	0	0	0	34.3	0	9.5	47.2
**156**	3	1	18	562	62	100	0.16	12.7	0	0	34.3	0	0	0	0	0	0	0	0	0	0	0	9.5	50.2
**157**	3	1	18	561	111	181	0.00	0	0	0	0	0	47	0	0	0	0	0	0	0	0	0	9.5	47.2
**158**	3	1	18	516	116	117	0.00	12.7	0	0	0	0	0	34.3	0	0	0	0	0	0	0	0	9.5	49.6
**159**	3	1	18	552	0	0	0.00	12.7	0	0	0	0	0	0	0	0	0	0	0	0	0	34.3	9.5	-
**160**	3	2	6	545	14	21	0.00	12.7	0	34.3	0	0	0	0	0	0	0	0	0	0	0	0	9.5	28.8
**161**	3	2	6	551	21	32	0.00	0	12.7	34.3	0	0	0	0	0	0	0	0	0	0	0	0	9.5	26.2
**162**	3	2	6	548	23	35	0.00	0	12.7	0	0	0	0	0	0	0	0	0	0	0	34.3	0	9.5	25.4
**163**	3	2	6	563	13	19	0.00	12.7	0	0	34.3	0	0	0	0	0	0	0	0	0	0	0	9.5	26.6
**164**	3	2	6	550	49	72	1.16	0	0	0	0	0	47	0	0	0	0	0	0	0	0	0	9.5	16.0
**165**	3	2	6	552	32	47	1.18	12.7	0	0	0	0	0	34.3	0	0	0	0	0	0	0	0	9.5	31.5
**166**	3	2	6	562	60	91	0.06	12.7	0	0	0	0	0	0	0	0	0	0	0	0	0	34.3	9.5	32.4
**167**	3	2	12	556	45	71	0.15	12.7	0	34.3	0	0	0	0	0	0	0	0	0	0	0	0	9.5	39.2
**168**	3	2	12	557	47	79	0.35	0	12.7	34.3	0	0	0	0	0	0	0	0	0	0	0	0	9.5	30.0
**169**	3	2	12	551	53	84	0.00	0	12.7	0	0	0	0	0	0	0	0	0	0	0	34.3	0	9.5	33.0
**170**	3	2	12	557	28	42	0.00	12.7	0	0	34.3	0	0	0	0	0	0	0	0	0	0	0	9.5	26.6
**171**	3	2	12	558	85	133	0.00	0	0	0	0	0	47	0	0	0	0	0	0	0	0	0	9.5	23.1
**172**	3	2	12	553	92	149	0.00	12.7	0	0	0	0	0	34.3	0	0	0	0	0	0	0	0	9.5	35.0
**173**	3	2	12	552	98	158	0.00	12.7	0	0	0	0	0	0	0	0	0	0	0	0	0	34.3	9.5	39.8
**174**	3	2	18	556	66	109	0.06	12.7	0	34.3	0	0	0	0	0	0	0	0	0	0	0	0	9.5	41.9
**175**	3	2	18	552	76	119	0.06	0	12.7	34.3	0	0	0	0	0	0	0	0	0	0	0	0	9.5	40.2

**176**	3	2	18	541	87	148	0.00	0	12.7	0	0	0	0	0	0	0	0	0	0	0	34.3	0	9.5	49.1
**177**	3	2	18	561	59	98	0.06	12.7	0	0	34.3	0	0	0	0	0	0	0	0	0	0	0	9.5	35.1
**178**	3	2	18	556	113	187	0.06	0	0	0	0	0	47	0	0	0	0	0	0	0	0	0	9.5	43.8
**179**	3	2	18	546	127	213	0.00	12.7	0	0	0	0	0	34.3	0	0	0	0	0	0	0	0	9.5	42.4
**180**	3	2	18	549	68	161	0.00	12.7	0	0	0	0	0	0	0	0	0	0	0	0	0	34.3	9.5	58.0
